# Peer Review of “Exploring the Reasons for Low Cataract Surgery Uptake Among Patients Detected in a Community Outreach Program in Cameroon: Focused Ethnographic Mixed Methods Study”

**DOI:** 10.2196/39041

**Published:** 2022-06-09

**Authors:** Alexandros Argyriadis

**Affiliations:** 1 Frederick University Nicosia Cyprus

**Keywords:** ophthalmologic surgical procedures, access to health care, ophthalmology, patient-centered care, ethnography, health knowledge, attitudes, practice

*This is a peer-review report submitted for the paper “Exploring the Reasons for Low Cataract Surgery Uptake Among Patients Detected in a Community Outreach Program in Cameroon: Focused Ethnographic Mixed Methods Study.”*

## Round 1 Review

### General Comments

This paper [1] studies the reasons for the low cataract surgical uptake among patients detected in a community outreach program in Cameroon, and it can be characterized as innovative as the research on that time-place context is limited. Moreover, this is an ethnographic approach that comes to fill the research gap in a huge database of quantitative data. There are some qualitative efforts that discuss the access of citizens in health care structures [2] but the issue of cataract has not been studied in terms of prevention and health promotion. However, the article contains both quantitative and qualitative data, and this is a positive aspect that endures the whole research project. It is evidence-based, and the authors offer the opportunity to the scientific community to have access to their database, if asked. The fact that this project is not only theoretical but also it can contribute to the quality of everyday life in Cameroon is evident by the funding that the research team received. They engaged the local community and institutions to the awareness of this issue and that is one of the main ingredients of the ethnographic methodology. This study has brought to light the potential of ethnography in uncovering the challenges faced by underprivileged populations in accessing cataract surgery and the related complexity that underpins the considerations in improving uptake of cataract surgery in low-resource and culturally inclined settings. It also shows that the patient-reported barriers to cataract surgery of those attending eye clinics (in this case for hospital-based studies) may not necessarily be the experience reflecting the communities they come from. Our results also provide useful insights regarding the planning of advocacy and other public health services.

Hence, οn the one hand, in the case of the methodological selection and on the other hand in the selection of the specific topic to be investigated, we can say that this is a very successful project, which would be of great interest if it expands to other areas where similar difficulties are identified. In the Community Health Care context, one of the most important issues discussed among experts is health promotion and the prevention of diseases of specific population groups characterized by risk factors. As there has been such a huge outbreak of awareness programs for the COVID-19 pandemic crisis, it would be useful to extend similar programs to other diseases that affect the health of communities over time [3].

The article concludes with the results that cost and fear were the main barriers to cataract surgery compounded by a strong belief in traditional medicine and superstition. These results apply to settings reliant on hospital-based delivery models with a disintegrated eye care delivery from the public health strategy and with little or no health coverage. Finally, the authors recognize the research implications and they come up with recommendations for further research.

### Specific Comments

#### Major Comments

1. There are no major amendments needed.

#### Minor Comments

1. It would be interesting if there are any other articles that mention this problem and can be added in the manuscript.

2. Moreover, the eye care delivery in Cameroon is presented only from the financial aspect. It would be interesting if the authors could add some other demographic or educational and cultural factors that affect the access to health care.
